# On the threshold - evaluation of variability in effects of acupuncture in a gender perspective

**DOI:** 10.1186/1749-8546-5-32

**Published:** 2010-09-04

**Authors:** Iréne Lund, Thomas Lundeberg

**Affiliations:** 1 Department of Physiology and Pharmacology, Karolinska Institutet, Stockholm, Sweden; 2 Foundation for Acupuncture and Alternative Biological Treatment Methods, Sabbatsbergs Hospital, Stockholm, Sweden

## Abstract

Variable results of pain alleviation in response to acupuncture have been reported, complicating its interpretation. Sources of variability are probably multi-factorial, including the contribution of gender related effects. Gender related variation in perceived pain has been discussed frequently, but documented effects of acupuncture referring to gender are sparse. Furthermore, factors such as operationalisation of the outcome variable and the statistical method for evaluation could also be sources of variability. When pain is regarded as subjective, the produced data should be treated as ordinal. The rank-based method by Svensson, taking the non-metric qualities of the ordinal data into account as well as the variability at the group and the individual level, is therefore an alternative. The present commentary aims to (1) evaluate changes in electrical sensory thresholds and electrical pain thresholds after low frequency electro-acupuncture separately in healthy women and men; (2) introduce and exemplify the method by Svensson in a user-friendly approach. To analyze the systematic patterns of change in thresholds, indicating evidence of treatment on a group level, the relative position (RP) and relative concentration (RC), were measured. The variation related to the individual, the relative rank variation (RV) was also measured. The results were divergent between women (*n *= 23) and men (*n *= 22), i.e. unchanged sensory threshold after acupuncture at the group level in women while changed in men. The assessed pain threshold after acupuncture on the other hand was changed towards higher levels in women and unchanged in men. The individual variation was apparent in both women and men but larger in women. For statistical analysis of the variability for both group and individual related effects, the rank-based method by Svensson could be used. The present study indicates that evaluation of sensory and pain threshold response should be analysed separately in women and men.

## Introduction

Some clinical trials that compared acupuncture with no treatment or other treatment modalities have been reported [1-6]. However, variability in the study results [7,8] makes the interpretation difficult [9-11]. Some positive results were mistakenly attributed to placebo [12].

Variability in acupuncture treatments of pain is multi-factorial, such as clinical conditions, treatment strategies, study designs, outcome variables, instrument used and gender. Gender differences in pain evaluation have been discussed [13] but documentation of gender related responses to acupuncture is scarce. Possible gender differences in response to transcutaneous electrical nerve stimulation (TENS) and vibration have been discussed [14,15].

The method used for statistical analysis, based on the operationalisation of the outcome variable like assessed pain intensity and its indicators, is important for the interpretation of the final results [16]. Pain is a complex, subjective and personal experience [17] with uncertain proportionality to nociception. Therefore, evaluation of perceived pain is based on self-reports using specific scales, questionnaires or instruments based on the psychophysical methodologies such as perceived thresholds for sensation and pain [18]. As pain is subjective, the data of pain have non-metric ordinal properties. The ordinal data do not indicate the magnitude and distance between categories of the assessment instrument [19,20]. Hence, statements such as 'twice as much' on a pain rating scale and percentage change are inappropriate [16]. However, it is still controversial whether subjective variables such as pain are equidistant with linear (metric) properties [21] and will have implications for the evidence based decisions and choice of recommendations of pain treatment.

A quite recently developed statistical method, suitable for data from scale assessments with only the order known, could be used be used to evaluate changes, inter- and intra-observer agreements [22,23]. The method takes the non-metric properties of the ordinal data into account *without *pre-defined assumption of distribution of the data other than the ordered structure, and is applicable to the raw data as it does not matter if they have linear, ordinal or dichotomy properties. Thus the results are valid and reliable for all types of ordered data even in small samples. Furthermore, the method gives the possibility of evaluating individually related changes separately from the systematic group related changes. When the objective of a study is evaluation of change, the interpretation of the systematic changes linked to the group effects gives support for evidence of the treatment, while the measure of individual variability illustrates the range of individual response.

## Threshold assessment study

In order to demonstrate how ordinal data can be analysed at both group and individual levels, we used an example to illustrate the changes of assessed thresholds in response to electro-acupuncture in participants of both genders.

### Participants

Students at the Karolinska Institutet who reported to be healthy participated in the study after their informed consent. The study protocol was approved by the Ethics Committee of Karolinska Hospital (dnr 01-169).

### Assessment

**Figure 1:**
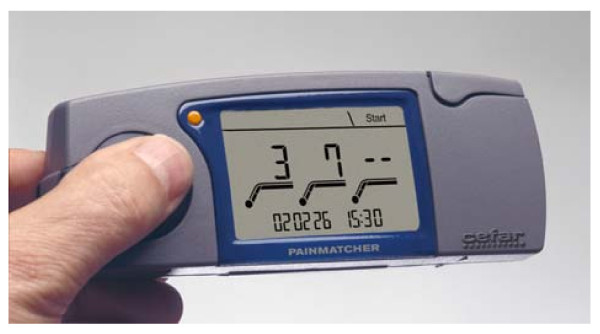
**The PainMatcher instrument used for assessments of EST and EPT**.

PainMatcher instrument (Pain-Matcher^®^, Cefar Compex AB, Sweden) [24,25] was used for threshold assessments (Figure 1). The instrument produced electrocutaneous stimulation with constant (15 mA) current through two electrodes at a frequency of 10 Hz compensated for variations in skin resistance up to 13 kΩ. The stimulation intensity increased along with the increase of the pulse duration in increments of 4 μs in a total of 99 steps (4 μs-396 μs). The subject released the fingers from the electrodes when the intended threshold was perceived. The assessed electrical sensory threshold (EST, i.e. the least perceived stimulation described as a paresthesia-like sensation) and the electrical pain threshold (EPT, i.e. the least stimulation leading to the first perception of pain distinct from unpleasantness) were recorded. The threshold assessments were guaranteed not to cause tissue damage at any level. The instrument was tested 3-4 times with all subjects sitting in a relaxed position for 10 minutes before the actual experiment.

### Electro-acupuncture

Low frequency electro-acupuncture, EA, (2 Hz) was used (Acus 1, Cefar Compex AB, Sweden) with distributed current in alternating pulses (180 μs, 0-12 mA). The electrodes were applied to the sterile disposable needles (30 mm×0.30 mm, Hegu Xeno, Hegu Svenska AB^®^, Sweden) placed at the acupoints ST36 and ST38 after *deqi *sensation was elicited. Acupoints ST36 and ST38 were selected because they are distant from the hand with minimal 'spinal segmental effects'. The current amplitude was increased until muscle contractions were seen and adjusted to below the level of perceived unpleasantness throughout the stimulation period of 20 minutes. Four threshold assessments were performed: (1) before EA, (2) during EA, (3) after EA for 20 minutes and (4) 10 minutes after ended EA.

### Statistical analysis

The age of the participants was presented as mean and standard deviation (SD). The data of the assessed EST and EPT were ordinal and hence presented as the median, range and interquartile range (IQR) of the PM values.

The pattern of changes in the paired data of EST and EPT, before and ten minutes after EA, were shown in square contingency tables, and in scatter plots respectively. The main diagonals in the contingency tables, consisting of grey-shaded cells, and of dotted lines in the scatter plots, indicates no change in the respective threshold from the one occasion to the other. The proportions of participants with increased, unchanged and decreased thresholds values after versus before EA were calculated, as were the 95% confidence intervals (95% CI), in proportions of changed threshold levels between the two independent groups of women and men. The hypotheses of no change in assessed EST and EPT comparing values before and after EA were analyzed by the non-parametric sign test. For a further and more detailed evaluation of the variability (both systematic and individual changes) of EST and EPT levels, we used the rank-based statistical analysis method by Svensson [23,26,27]. Its principal formulae are given below and are further on demonstrated within the calculations of applied data from the women's EST before versus after EA *P*-values less than 0.05 were considered statistically significant.

STATISTICA 8.0 (StatSoft^® ^Scandinavia AB, Sweden) was used for descriptive statistics and analysis with the Sign test. For calculation of confidence interval of proportional change in the two groups the Computer software for Confidence Interval Analysis (CIA) [28] was used and for calculation of with the Svensson method its free software [27] was used.

## Description of rank-based statistical method by Svensson

### Systematic variability and heterogeneity

**Figure 2:**
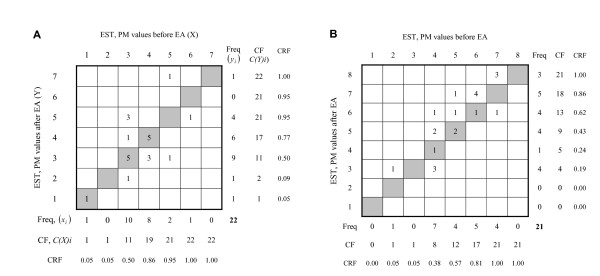
**Contingency tables of paired EST assessments in a) healthy women (*n *= 22) and b) healthy men (*n *= 21), before EA, X, and after EA, Y**. Freq = Frequency of recorded PM values, marginal frequencies, before EA and after EA; CF = Cumulative frequency of the marginal frequencies before and after EA (C(X)i; C(Y)i); CRF = Cumulative relative frequencies.

A systematic change, related to the group, appears as different marginal frequency distributions, heterogeneity, of the assessments seen in the contingency tables and plots (Figure 2a-b). Two types of systematic change in position and concentration in the two sets of paired data are calculated, the relative position (RP) and the relative concentration (RC). The measure of RP is calculated as the difference between the estimated probabilities of the marginal distributions before EA (X) being shifted towards decreased and towards increased values relative to the marginal frequency distributions after EA (Y), *P(X < Y)-P(Y < X)*.

In the formulae below the number of individuals is denoted *n *and the number of categories of the scale is denoted *m*, then

(1) $$\begin{array}{l} \text{RP}=p_{xy}-p_{yx}\\ \text{where }p_{xy}=\frac{1}{n^{2}}\sum_{i=1}^{m}\left[y_{1}•C{\left(X\right)}_{i-1}\right]\text{ and }p_{yx}=\frac{1}{n^{2}}\sum_{i=1}^{m}\left[x_{1}•C{\left(Y\right)}_{i-1}\right] \end{array}$$

The RC is calculated as the difference between the probabilities of the assessed threshold values being more concentrated to the central PM values between 1 to 99 than to the peripheral parts of the possible PM values after EA than before EA and vice versa *P *(*X*_1 _<*Y*_*k *_<*X*_0_) - *P*(*Y*_1 _<*X*_*k *_<*Y*_0_)

then

(2) $$\text{RC}=\frac{1}{Mn^{3}}\sum_{i=1}^{m}\left\{y_{\text{i}}•C{\left(X\right)}_{i-1}\left[n-C{\left(X\right)}_{i}\right]-x_{i}•C(Y)\left[n-C{\left(Y\right)}_{i}\right]\right\}$$

where *M *= minimum value of (*p_xy _*- *p_xy_*^2^) and (*p_yx _*- *p_yx_*^2^) provided 0 < (*p_xy _*and *p_yx_*) < 1

Possible values of RP and RC range from -1 to 1. A positive RP value indicates increased thresholds on the second occasion and the contrary holds for a negative RP value. A positive value of RC indicates that the assessed values are more concentrated towards the central parts of the scale on the second occasion than the first, while negative RC values reflects an adjustment towards the ends of the scale.

**Figure 3:**
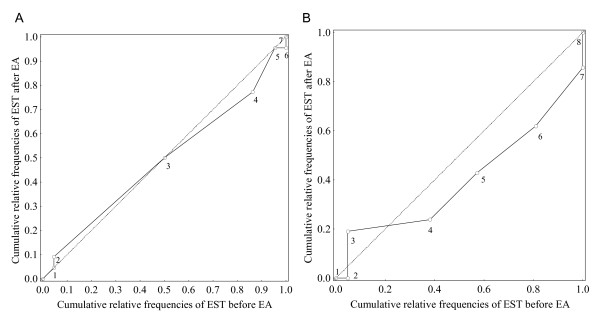
**ROC curves demonstrating the cumulative relative frequencies of EST before and after EA in a) women, *n *= 22, EST ranging from 1 to 7, and in b) men, *n *= 21, EST ranging from 1 to 8**. From the ROC curve in Figure 3a it appears that the EST was assessed 4 in 86% of the women before EA and in 77% of the 22 women after EA. It is also obvious that the median EST was 3 (50% of the 22 women) both before and after EA. The ROC is nearly close to the main diagonal indicating that there was no systematic change among the women in the assessed EST before compared to after EA. The ROC curve in Figure 3b indicates systematic change both in position and concentration in assessed EST among the men.

The presence of systematic change is graphically illustrated with plotting the two sets of cumulative relative proportions of the marginal frequency distributions in the contingency tables against each other in a sort of a relative operating characteristic (ROC) curve starting at the (0,0) point with a main diagonal representing no change (Figure 3a-b). A non-zero RP value means that the ROC curve deviates from the diagonal indicating the presence of a systematic change. In this case a positive RP is evident by a ROC curve deviating below the main diagonal. A systematic change of concentration is characterized by an S-shaped ROC-curve indicating that one set of the assessments is concentrated to a certain part, central or peripheral, of the scale compared to the other set. A lack of systematic change is indicated by the ROC curve close to main diagonal. In this case, the evaluation of the systematic changes reflects the treatment efficacy on the entire group.

### Individual variation

A presence of individual variation in the pattern of change, *not *explained by a systematic change, is evident from pairs of observations in the contingency tables of EST or in the scatter plots of the EPT data when dispersed from the expected pattern of change. To estimate the contribution of the individual variation to the pattern of change, firstly an augmented mean rank procedure is used. The augmented ranking is defined by the observations in the (*i*, *j*)^th ^cell of the contingency table according to values before EA (X) as

(3) $${\bar{R}}_{ij}^{\left(X\right)}=\sum_{k=1}^{i-1}\sum_{l=1}^{m}x_{kl}+\sum_{l=1}^{j-1}x_{il}+\frac{1}{2}(1+x_{ij})$$

for 1 ≤ *i, j*≤ *m *where the x_ij _is the *ij*^th ^cell frequency. The corresponding augmented mean ranks according to values after EA, Y, ${\bar{R}}_{ij}^{\left(Y\right)}$ were similarly defined. The squared augmented mean rank differences define the measure of the individual part of in the pattern of change and is denoted the relative rank variance,

(4) $$\text{RV}=\frac{6}{n^{3}}\sum_{i=1}^{m}\sum_{j=1}^{m}x_{ij}\Delta {\bar{R}}_{ij}^{2}$$

The measure of RV ranges from 0 to 1. The higher the values of RV, the more dispersed are the observations from the expected pattern of change [23]. Strong evidences of additional individual changes, high RV values, indicate that individually designed interventions like acupuncture would be preferable.

## Findings of the threshold assessment study

A total of 23 women aged 27.7 (6.4) years and 22 men aged 28.6 (6.5) participated in the study. All subjects fulfilled the protocol with one women and one man failing to report the EST results.

### Change of EST

The median EST was reported by the women as 3 (range 1-6; IQR 1) before and 3 (range 1-7; IQR 1) after EA respectively. EST increase was reported by 5 (23%) of the 22 women, unchanged by 11 (50%) and a decreased EST by the remaining 6 (27%) women.

The assessed median EST in the men were 5 (range 2-7; IQR 2) before and 6 (range 3-8; IQR 2) after EA respectively. EST increase was reported by 13 (62%) of the 21 men, unchanged by 4 (19%) and decreased by 4 (19%). The difference in proportions in women and men who reported EST increase was -39% (95%CI, -23% to -62%), i.e. a greater EST increase was reported by the men than the women after EA.

The pattern of change in EST, shown as the frequency of the reported PM values in the contingency tables, is denoted X before and denoted Y after EA (Figure 2a-b). The grey shaded diagonal cells in the contingency tables represent unchanged EST. Figure 2a shows that one woman reported a PM value of 1 as her EST before and after the EA. Similarly, five women reported PM value as 3 before and after and another five women reported their unchanged PM value as 4 before and after EA.

### Applying the Svensson method

#### Systematic, group related, changes

The evaluation of possible systematic changes is based on the heterogeneity of marginal frequency distributions from the pairs of assessments before and after EA (*X_i_*, *Y_i_*) respectively. Let *x_i _*and *y_i _*denote the frequencies of the *i*^th ^PM values of the individuals and *C(X)i *and *C(Y)i *denote the cumulative frequencies of the *i*^th ^PM value of the two sets of marginal frequency distributions, *X *and *Y*. First the measure of systematic change in position, denoted relative position (RP) was calculated according to the formula **(1) **in the statistical section

$$\begin{array}{l} \text{RP}=p_{xy}-p_{yx}\text{ where}\\ p_{xy}=\frac{1}{n^{2}}\sum_{i=1}^{m}\left[y_{1}•C{\left(X\right)}_{i-1}\right]\text{ and }p_{yx}=\frac{1}{n^{2}}\sum_{i=1}^{m}\left[x_{1}•C{\left(Y\right)}_{i-1}\right] \end{array}$$

Applied to the distribution of marginal frequencies of the EST data reported by the women in Figure 2a and Table 1,

**Table 1:** Distribution of marginal frequencies of PM values of assessed EST according to Figure 2a

PM values	1	2	3	4	5	6	7
Frequencies before EA *(x_i_)*	1	0	1	8	2	1	0
Cumulative frequencies before EA, *C(X)i*	1	1	11	19	21	22	22
Frequencies after EA *(x_i_)*	1	1	9	6	4	0	1
Cumulative frequencies after EA, *C(Y)i*	1	2	1	17	21	21	22

$$p_{xy}=\frac{1}{22^{2}}\left[1\times 1+9\times 1+6\times 11+4\times 19+0\times 21+1\times 22\right]=0.360;$$

and

$$p_{yx}=\frac{1}{22^{2}}\left[0\times 1+10\times 2+8\times 11+2\times 17+1\times 21+0\times 21\right]=0.337;$$

                          RP = 0.023.

Using the same pair of data the evaluation of the other type of systematic change in EST, we calculated relative concentration (RC) with formula **(2)**, i.e.

$$\text{RC}=\frac{1}{Mn^{3}}\sum_{i=1}^{m}\left\{y_{\text{i}}\times •C{\left(X\right)}_{i-1}\left[n-C{\left(X\right)}_{i}\right]-x_{i}•C(Y)\left[n-C{\left(Y\right)}_{i}\right]\right\}$$

where *M *= minimum value of (*p*_*xy *_- *p*_*xy*_^2^) and (*p*_*yx *_- *p*_*yx*_^2^) provided 0 < (*p_xy _*and *p_yx_*) < 1

Correspondingly,

$$\begin{array}{l} \text{RC}=\frac{1}{0.223\cdot 22^{3}}\times (\text{1}\times \text{1}\times \text{21}+\text{9}\times \text{1}\times \text{11}+\text{6}\times \text{11}\times \text{3}+\text{4}\times \text{19}\times \text{1}+0\times \text{21}\times 0+\text{1}\times \text{22}\times 0-\\ 0\times \text{1}\times \text{2}0+\text{1}0\times \text{2}\times \text{11}+\text{8}\times \text{11}\times \text{5}+\text{2}\times \text{17}\times \text{1}+\text{1}\times \text{21}\times \text{1}+0\times \text{21}\times 0);\\ \text{RC}=\frac{394-715}{0.223\cdot 22^{3}}=-0.135 \end{array}$$

The RP value was small, 0.02 and close to zero while there was a certain change in concentration, RC-0.14, towards the peripheral ends of the scale categories used. However, no evidence of significant systematic changes in assessed EST was found in the applied example; RP (95%CI, -0.24 to 0.29) and RC -0.14 (95%CI, -0.30 to 0.03) since the confidence interval in both cases did cover the zero in the calculated systematic changes.

The corresponding results of assessed EST in the group of men were RP 0.20 (95%CI, 0.02 to 0.38) and RC -0.31 (95%CI, -0.59 to -0.03), indicating a systematic change towards higher EST and a spread of the PM values towards the end of the scale after EA as compared with before EA. The systematic change of EST in position among the men, RP 0.20, indicates a 20% greater chance that the EST is judged higher after EA compared with before as the opposite in a similar sample of healthy men. Correspondingly, the RC -31 indicates a 31% greater chance that the PM values of assessed EST among the men will be concentrated to the peripheral part of the scale after EA.

The values of RP and RC close to zero indicate a lack of systematic changes for the group and produced a ROC curve close to the main diagonal as shown in Figure 3a. A deviating ROC-curve indicates a systematic change in position where the size of deviation is related to the systematic change measured by RP and the S shape of the ROC curve is a sign of systematic change in concentration of the assessments (Figure 3b).

**Figure 4:**
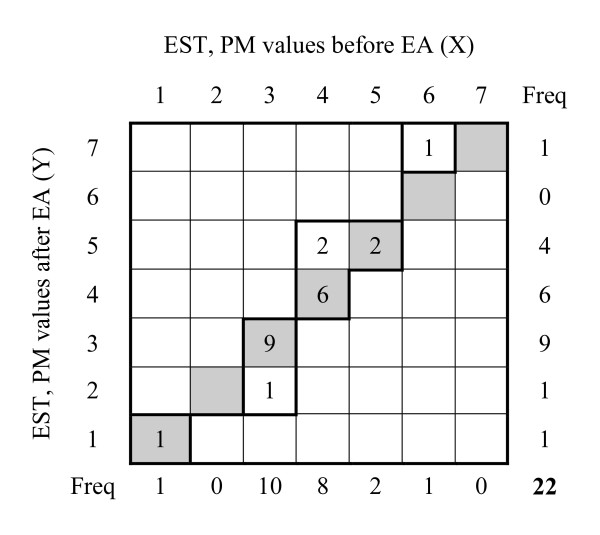
**The *expected *pattern of change in EST according to the rank-transformable pattern of change (RTPC) referring to the pure systematic change common to the group based on data in contingency table, Figure 2a, where all individuals have kept their ordering relative to each other**.

To evaluate the pattern of *pure *systematic change without any source of individual variation in the paired observations (i.e. the *expected *pattern of the systematic change), we used the rank-transformable pattern of change (RTPC) to pair off the two sets of marginal frequencies where all individuals kept their ordering relative to each other but may have changed the category (Figure 4).

According to Figure 2a, the first observation in category 1 (PM value 1) before EA is paired with the first observation in category 1 (PM value 1) after EA and the first following observation in category 3 (PM value 3) before EA is paired with the observation in category 2 (PM value 2) after EA. The following nine observations in category 3 (PM value 3) before EA is paired with the nine observations in category 3 after EA and so on. Hence, there will be one pair (1,1) and one pair (3,2), nine pairs (3,3) and six pairs (4,4). According to the calculated RTPC for the women in this study, the expected EST after EA is unaffected by acupuncture stimulation in the ST36 and ST38 except for four of the women. According to this pattern one were supposed to have reduced EST, (from 3 to 2), and three of them that were supposed to report increased EST, from 4 to 5 and 6 to 7 respectively, after EA as compared with before EA.

#### Individual variation

The existence of heterogeneity in the marginal frequencies in observed data, i.e. indications of systematic changes related to the group, does not entirely explain the pattern of change in paired assessments. Often there is an individual source of heterogeneity in the results additionally to the systematic changes in studies evaluating treatment of pain or other subjective variables. When *observed *values (Figure 2a) are dispersed from the *expected *pattern of change at the group level, RTPC (Figure 4), it is an indication of individual variation. For example four individuals (3+1) assessed their EST as 4 and 5 before EA that was shifted to 3 after EA (Figure 2a). The expected assessments based on the result of the group were PM value 3 both before and after EA (Figure 4).

**Figure 5:**
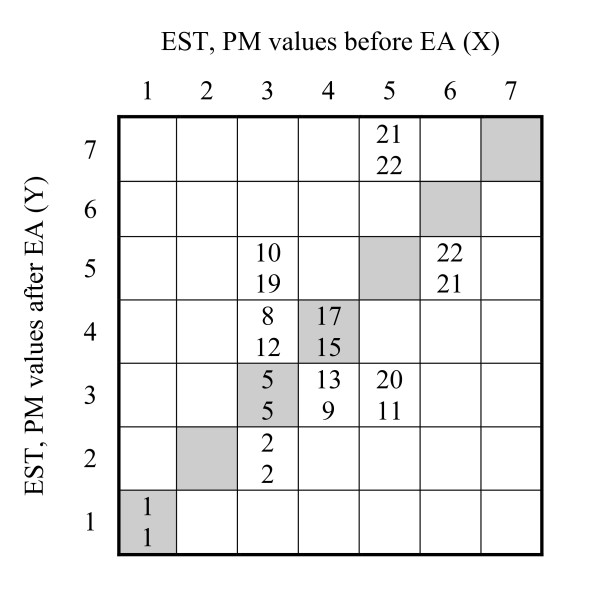
**Pair of augmented mean ranks for observations in Figure 2a**. Each cell shows above and ${\bar{R}}_{ij}^{\left(Y\right)}$ below.

The evaluation of individual changes in the applied threshold assessments are based on a ranking procedure in which the pairs of assessments are transformed into ranks, tied on the cells (Figure 5), the augmented ranks. The augmented ranking procedure according to formula **(3)**

${\bar{R}}_{ij}^{\left(X\right)}=\sum_{k=1}^{i-1}\sum_{l=1}^{m}x_{kl}+\sum_{l=1}^{j-1}x_{il}+\frac{1}{2}(1+x_{ij})$ applied to the data of the women's EST is for example, according to Figure 2a, = 11+3+0.5(1+5) = 17 and ${\bar{R}}_{4,4}^{\left(Y\right)}=11+1+0.5(1+5)=15$. Furthermore, the pairs with EST level (1,1) and (3,2), but also the five pairs with EST (3,3), share the same mean rank value according to the distribution before and after EA (Figure 2a). The three pairs of data with the EST (3, 5) share the ranks 9, 10, 11 at the first assessment with the mean rank 10. At the second assessment these three observations share the ranks 18, 19, 20 that is the mean rank 19 (Figure 5) and so on. A further analysis of the joint distribution of the paired data (Figure 5) is required for taking account of the individual variation in the pain data.

The measure of the individual changes, the relative rank variance is then defined by the square of the mean rank differences as in formula **(4) **where $\text{RV}=\frac{6}{n^{3}}\sum_{i=1}^{m}\sum_{j=1}^{m}x_{ij}\Delta {\bar{R}}_{ij}^{2}$

Then, according to Figures 2a and 5

$$\begin{array}{l} \text{RV}=\frac{6}{22^{3}}1{\left(12-8\right)}^{2}+3{\left(19-10\right)}^{2}+3{\left(13-9\right)}^{2}+\\ 5{\left(17-15\right)}^{2}+1{\left(20-11\right)}^{2}+1{\left(22-21\right)}^{2}+1{\left(22-21\right)}^{2};\\ \text{RV}=0.231 \end{array}$$

The pair of rank values for the four individual that have the mean rank differences of 9 respectively, means that these individuals contribute to a large extent to the heterogeneity in the group regarding assessed EST before and after EA as indicated by the significant measure of RV 0.23 (95%CI, 0.00 to 0.51). However, among the men the individual variation was considered small and negligible RV 0.03 (95%CI, 0.00 to 0.08) indicating that the individuals among the men agreed in their opinion of changed EST.

#### Systematic and individual changes in assessed EPT

**Figure 6:**
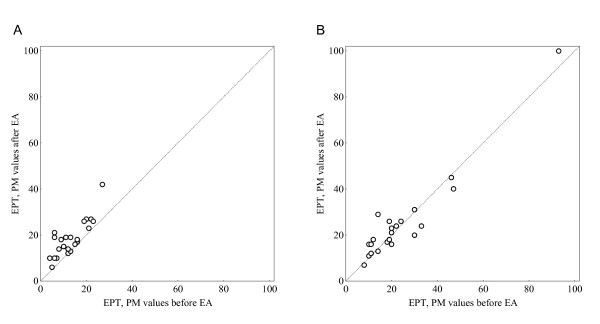
**Scatterplots of individual paired assessments on EPT before and after EA in (a) women (*n *= 23) and (b) men (*n *= 22)**.

Among the women, the assessed median EPT was 12 (range 4-27; IQR 13) before and 18 (range 6-42; IQR 10) after EA. Twenty-one (21) of the 23 women (91%) reported the EPT as increased while two of them (9%) reported it as unchanged after EA. This change was considered significant, *P *< 0.001 (Figure 6a).

The median EPT in the group of men was 19 (range 8-93; IQR18) before and 20 (range 7-99; IQR 10) after EA respectively. Among the 22 men, 13 of them (59%) reported an increase of the EPT and the remaining 9 (41%) a decrease (Figure 6b). The difference of proportional changes between women and men in EPT was 33% (95% CI, 10% to 56%), i.e. the increase in assessed EPT was greater among the women than then in the men after EA.

**Figure 7:**
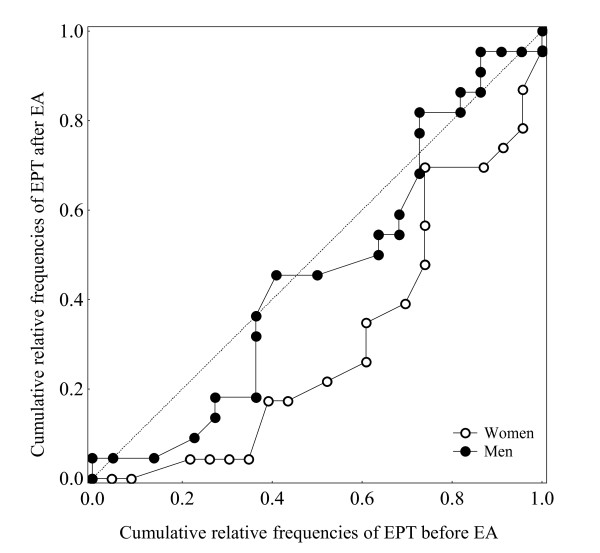
**The ROC curve of systematic changes in EPT based on marginal frequencies of assessed EPT before and after EA in women (*n *= 23) and men (*n *= 22)**.

The findings of systematic changes in EPT were also confirmed by the use of the Svensson method showing a systematic change in position, RP 0.39 (95% CI, 0.23 to 0.56) in the women but not in the men, RP 0.09 (-0.06 to 0.24). The measure of relative concentration was similar in the both groups, RC 0.16 (95% CI, -0.06 to 0.38) indicating a change of the PM values towards more central part of the scale, but without evidence of significant change (Figure 7).

The change referred to the individual variation was large with a wide CI in the group of women, RV 0.24 (95%CI 0.00 to 0.49) while it was smaller but obvious also among the men, RV 0.13 (95%CI 0.00 to 0.30).

## Discussion

The exemplified study showed that changes of EST and EPT after EA vary in the groups of healthy women and men. In the female group, the EST was unchanged at the group level and was systematically changed among the men in both positions (increased) and in concentration (towards the peripheral part of the scale) after EA. On the other hand, the EPT results showed the opposite pattern of change where the women responded with a systematic change towards increased EPT after EA while the men remained unchanged. The group related effect among the women was supported by the fact that 21 of the 23 responded with increased EPT after EA. Apart from this group related change, the women were dissentient on the number of categories when the threshold was increased, i.e. the individual variability was obvious. Among the men an individual variation was also apparent. The responses to electro-acupuncture may differ between healthy subjects and patients with different pain conditions. However, the observed variability in the reported findings indicates that effects of EA should be evaluated separately in women and men. Moreover, the treatment effects of EA may lead to more trustworthy results when the treatment is individually designed.

Threshold assessments have been used in various studies of acupuncture applying different types of physical stimulation [29-31] with the objective of confirming hypoalgesic effects where an increase is attributed to activation of endogenous pain inhibitory systems [32]. The differences between women and men confirmed by the use of PM, for threshold assessments and evaluated with the Svensson method, are in line with our previous results of TENS, [14] and vibration [15]. The mechanisms of the gender differences in response to sensory stimulation such as acupuncture are unknown although biological, psychological and social factors are all likely to contribute to the differences [33,34]. Differences in sensitivity to electrical stimulation between women and men have also been discussed [35,36] and the dosage of acupuncture (e.g. stimulation intensity and duration) must be taken into consideration [37]. The assessed threshold levels are generally dependent on the status of the nervous system and the interaction between the sex hormones and the endogenous pain modulating systems [38].

The assessment of thresholds may be a valuable complementary instrument, both in clinical and research work, for the evaluation of pain as the pain threshold levels are supposed to change as a consequence of neural plasticity in long-term pain conditions [39]. Pain threshold assessments have also been suggested to act as indicators of treatment dosage [40].

Though challenging and difficult to assess and evaluate, the statistical evaluation of pain is of great importance to take into consideration. Otherwise important information could be missed, and the basic data for decision-making could be misleading. In the present study the applied statistical approach by Svensson [22,23,26,27] was applied for a further evaluation in addition to the tested hypothesis of no change by sign test and the proportional calculation of change of paired threshold assessments before and after acupuncture. The method was applied to ordinal data, but is also suitable for other types of data (e.g. equidistant and continuous data with linear properties). The advantage of statistical methods that do not require metric or other distributional properties of data are that the results are reliable and valid without restrictions and may also be used for small samples. Furthermore, the possibility of separating the pattern of change into both systematic and individual components is important in clinical work. The one component measuring the systematic effect, the RP and RC, concern what is relevant change on the group level and indicate evidence of treatment effects. The other component measuring the variability unexplained by the group concerns the individual variation, the RV, of the results and indicate the need to consider individually modified treatments like, for instance, the number of treatments, the "dosage" of treatment etc.

## Conclusion

The present study indicates that evaluation of sensory and pain threshold responses to acupuncture should be analysed separately in women and men. For statistical evaluation of the variability for both group and individual related effects, the rank-based method by Svensson could be used.

## Abbreviations

CI: confidence interval; CF: cumulative frequency; CRF: cumulative relative frequency; EA: electro-acupuncture; EST: electrical sensory threshold; EPT: electrical pain threshold; FREQ: frequency; IQR: interquartile range; PM: PainMatcher; RC: relative concentration; RP: relative position; RV: relative rank variance; RTPC: rank transformable pattern of change; ROC: relative operating characteristic; SD: standard deviation; ST: stomach; TENS: transcutaneous electrical nerve stimulation; Tot: total;

## Competing interests

The authors declare that they have no competing interests.

## Authors' contributions

TL conceived the idea of the manuscript. IL collected and analysed the data. Both authors contributed equally to the writing and finalising of the manuscript. Both authors read and approved the final version of the manuscript.
